# Supplementary material for: CCl4 emissions in eastern China during 2021–2022 and exploration of potential new sources
Source: Nat Commun. 2024 Feb 26;15:1725. doi: 10.1038/s41467-024-45981-x (PMC10897440; doi:10.1038/s41467-024-45981-x)
Supplement: Supplementary file 1 — Supplementary information [file 41467_2024_45981_MOESM1_ESM.pdf]

Supplementary Information

CCl_4 emissions in eastern China during 2021–2022 and exploration of potential new sources

Bowei Li[1], Jiahuan Huang[2], Xiaoyi Hu[1], Lulu Zhang[2], Mengyue Ma[1], Liting Hu[1], Di Chen[1], Qianna Du[1], Yahui Sun[1], Zhouxiang Cai[1], Ao Chen[3], Xinhe Li[1], Rui Feng[1], Ronald G. Prinn[4], Xuekun Fang[1,4*]

[1]College of Environmental & Resource Sciences, Zhejiang University, Hangzhou, Zhejiang 310058, China

[2]Wuxi Ecology Environment Monitoring and Control Center, Wuxi, Jiangsu 214000, China

[3]Environmental Health and Engineering department, Johns Hopkins University, MD 21211, United States

[4]Center for Global Change Science, Massachusetts Institute of Technology, Cambridge, MA 02139, United States

Supplementary Discussion

Supplementary Discussion 1: CCl_4 concentrations in eastern China

During the observation period, the average CCl_4 concentrations were 79.3 ± 14.3 ppt and 80.0 ± 14.6 ppt at ZJU and 76.8 ± 14.3 ppt and 77.9 ± 14.6 ppt at SHH in 2021 and 2022, respectively (Fig. 2a, Supplementary Supplementary Figure **3**). The concentrations were higher than the measurement in the four remote northern hemispheric stations (Mace Head (75.9 ± 0.23 ppt; 53.327°N, 9.904°W), Jungfraujoch (75.4 ± 0.29 ppt; 46.548°N, 7.985°E), Trinidad Head (76.0 ± 0.22 ppt; 41.054°N, 124.151°W), and Ragged Point (75.9 ± 0.26 ppt; 13.165°N, 59.432°W)) from the Advanced Global Atmospheric Gases Experiment (AGAGE; http://agage.mit.edu/) in 2021. Approximately 17% and 13% of the CCl_4 concentrations were higher than 90 ppt at ZJU and SHH, respectively, and ~6% and 5% of the CCl_4 concentrations were higher than 100 ppt at ZJU and SHH, respectively. These observations indicate the presence of substantial CCl_4 emissions in eastern China despite phasing out the production and consumption of CCl_4 for dispersive use since 2010. The four AGAGE stations used SIO-05 standard gas (Scripps Institution of Oceanography, USA[1]) calibration scale, while this study used Linde Gas, thus introducing a potential bias. Nevertheless, the differences between these standard gases are likely small[2-4], e.g., the ratio between CCl_4 concentrations determined with SIO-05 standard gas and Linde Gas was 1.02[4].

Using the robust extraction of baseline signals (REBS) method[5], approximately 30%

of the CCl_4 concentrations at ZJU (averaged as 93.9 ± 14.4 ppt) were identified as non-background concentrations, respectively. The background concentrations of CCl_4 from the REBS method at ZJU was 76.4 ± 5.7 ppt. The moderate frequency and high average of non-background CCl_4 concentrations observed at ZJU indicate that CCl_4 emissions occurred in eastern China during 2021–2022.

Since 2010, the CCl_4 concentrations measured at various regions in China were higher than the northern hemispheric background concentrations at the four stations in AGAGE, e.g., enhanced CCl_4 concentrations in Beijing (approximately 34 ppt in 2015)[6], Nanjing (approximately 32 ppt in 2018)[7], Lushan (approximately 94 ppt during 2011–2012)[8], Hebei (approximately 10 ppt in 2016)[4], Dongying (approximately 44 ppt in 2017)[9], Hangzhou (approximately 18 ppt during 2021–2022), and Shanghuang (approximately 17 ppt during 2021–2022). This suggests that despite the phasing-out of CCl_4 production and consumption for dispersive uses, CCl_4 emissions were substantial in China after 2010.

Supplementary Discussion 2: Cluster analysis of air masses arriving at the observation site

Using the Hybrid Single-Particle Lagrangian Integrated Trajectory Model (HYSPLIT) (http://www.arl.noaa.gov/ready/hysplit4.html)[10], the air masses reaching ZJU and SHH during the observation period were categorized into four and five trajectory clusters, respectively (Supplementary Supplementary Figure **6**). For ZJU, the average concentration of CCl_4 in Cluster #2 (79.8 ± 10.4 ppt) was higher ($p < 0.01$) than that

in Cluster #1 (76.8 ± 10.6 ppt), Cluster #4 (76.7 ± 10.8 ppt), and Cluster #3 (75.4 ± 8.2 ppt). Cluster #2 originated from Hebei province and passed over Shanghai and northern Zhejiang provinces. Although Cluster #1 also passed over Shandong and Jiangsu, as it originated at higher altitudes (1700–2000 m) than Cluster #2 (1000–1200 m), its CCl_4 concentration was significantly lower ($p < 0.01$). For SHH, Clusters #1 and #5 had significantly higher ($p < 0.05$) average CCl_4 concentrations (79.0 ± 8.6 ppt and 79.0 ± 12.4 ppt, respectively) than the other clusters.

Supplementary methods

Cluster analysis of air mass

To identify potential impacts of long-range sources, back trajectories of the sampled air masses were analyzed using the Hybrid Single-Particle Lagrangian Integrated Trajectory Model (HYSPLIT) (http://www.arl.noaa.gov/ready/hysplit4.html)[10]. The meteorological data used for running HYSPLIT were the Global Data Assimilation System (GDAS) dataset (3 h and 1-degree resolution; https://www.ready.noaa.gov/archives.php). The 120-h backward trajectories during the sampling period were calculated using HYSPLIT with the height set as 100 m above ground level and 1100 m above sea level at ZJU and SHH, respectively. Then, backward trajectories gained from HYSPLIT were analyzed based on the Euclidean distance clustering algorithm using the Geographical Information System (GIS)-based TrajStat software (http://meteothink.org/docs/trajstat/index.html) developed by Wang et al.[11] to distinguish different types of air mass. The equation for calculating Euclidean distance between trajectories is as follows[12]:

$$\mathrm{d}_{12} = \sqrt{\sum_{i=1}^{n}((X_1(i) - X_2(i))^2 + (Y_1(i) - Y_2(i))^2)} \quad (1)$$

where $X_1(Y_1)$ and $X_2(Y_2)$ represent the backward trajectories 1 and 2, respectively

Industrial sample collection

To ensure the stability of industrial samples during collection, transportation, storage, and analysis, this study used 3.2 L SUMMA stainless steel canisters (Entech

Instrument, Inc., Simi Valley, CA, USA) to store the samples. However, the canisters that store high-concentration pollutants often need to be cleaned repeatedly after the sample is analyzed, and yet there remains a risk of contaminating residues. Therefore, this study adopted different sample collection schemes for different concentrations of industrial waste gas. Before sample collection, a portable detector (PGM-7340, RAE Systems, CA, USA) was used to determine the concentration of total VOCs (TVOCs) at the chimney or workshop of the sampling enterprise. If the concentration of TVOCs was > 4 ppm (parts per million), a sampling gun (ZR-D03, Junray Instrument Co., Ltd., Qingdao, China; the concentration of TVOCs in the gun should be close to the ambient level before use) connected to a sampling pump (ZR-3520, Junray Instrument Co., Ltd., Qingdao, China) was used to collect whole air samples into the airbag. Then, an airtight syringe was used to extract a portion of the sample (the specific volume was determined by multiplying the volume of the canister by the ratio of 0.4 ppm to the concentration measured by the portable detector) from the airbag and transferring it to a SUMMA canister. Otherwise, samples were directly collected by SUMMA canisters fitted with particulate filters (47 mm, Whatman, Clifton, NJ, USA). In both cases, the position of the sampling inlet is as close as possible to the center of the discharge pipe.

Supplementary Table

Supplementary Table 1. Posterior emissions (Gg yr^{-1}) of CCl_4 in each province in eastern China

Province	2021		2022	
	Mean	std	Mean	std
Shanghai	0.18	0.02	0.14	0.01
Jiangsu	1.15	0.16	1.34	0.20
Zhejiang	1.25	0.09	1.28	0.08
Anhui	0.66	0.21	0.81	0.31
Fujian	0.72	0.52	1.26	0.63
Jiangxi	1.72	0.76	0.47	0.73
Shandong	0.96	0.76	2.11	0.77
Henan	0.39	1.06	0.77	1.69

Supplementary Table 2. Full names, abbreviations, and number of samples for each industry classified by national economic attribute

NO.	Full name	Abbreviation	Number of samples
1	Manufacture of rubber and plastics products	MRPP	105
2	Manufacture of general purpose machinery	MGPM	51
3	Manufacture of automobiles	MA	48
4	Manufacture of textile	MT	41
5	Manufacture of computers, communication and other electronic equipment	MCCO	41
6	Manufacture of metal products	MMP	34
7	Manufacture of electrical machinery and apparatus	MEMA	27
8	Printing and reproduction of recording media	PRRM	26
9	Manufacture of special purpose machinery	MSPM	24
10	Manufacture of raw chemical materials and chemical products	MRCM	20
11	Manufacture of medicines	MM	10
12	Processing of petroleum, coal, and other fuels	PPCF	4
13	Utilization of waste resources	UWR	4
14	Manufacture of railway, ship, aerospace and other transport equipment	MOTE	4
15	Manufacture of paper and paper products	MPPP	3
16	Manufacture of furniture	MF	3
17	Other*	OTHE	11

Note:

*Other: includes manufacture of liquor; beverages and refined tea; smelting and pressing of ferrous metals; manufacture of non-metallic mineral

Supplementary Table 3. The set-ups for the four inversions

Inversion	Baseline filtering method	Priori emission distribution
Inv-REBS-UniformPrior	REBS	Uniformly distributed in eastern China, and population-proxy distributed in other areas
Inv-AGAGE-UniformPrior	AGAGE	Uniformly distributed in eastern China, and population-proxy distributed in other areas
Inv-REBS-PopPrior	REBS	Population-proxy distributed
Inv-AGAGE-PopPrior	AGAGE	population-proxy distributed

Supplementary Figure

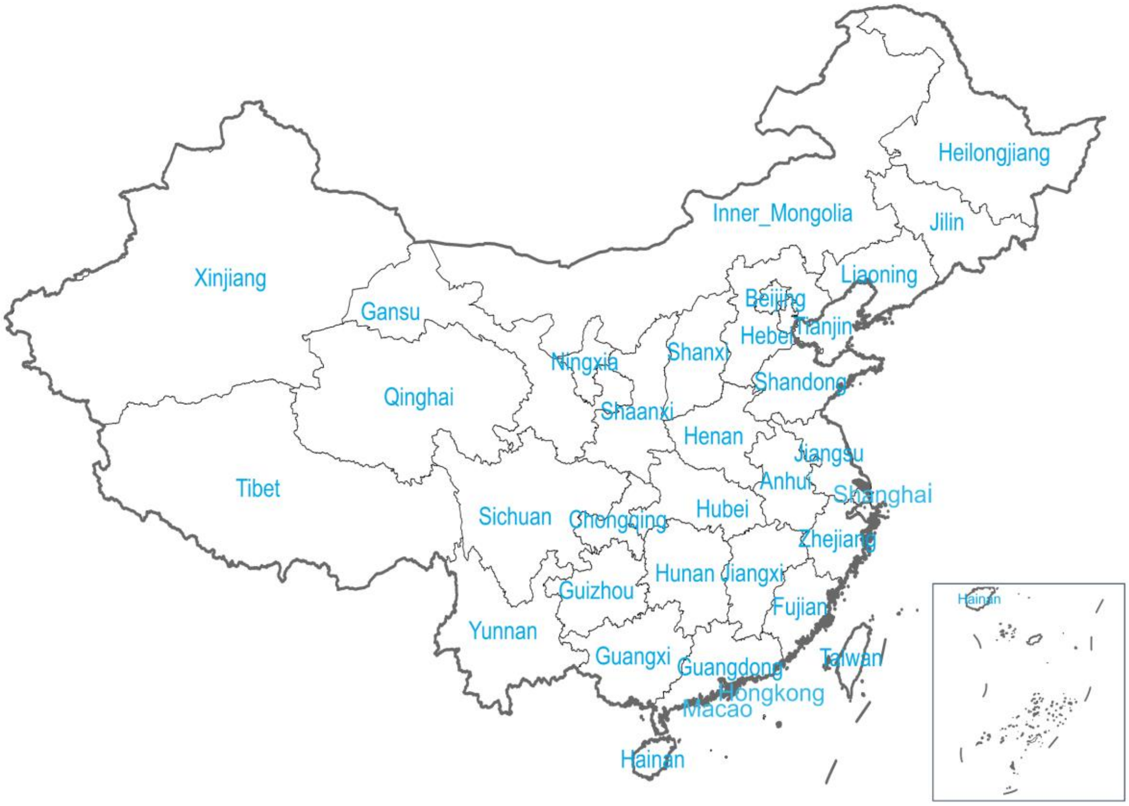

Supplementary Figure 1. Provinces in China.

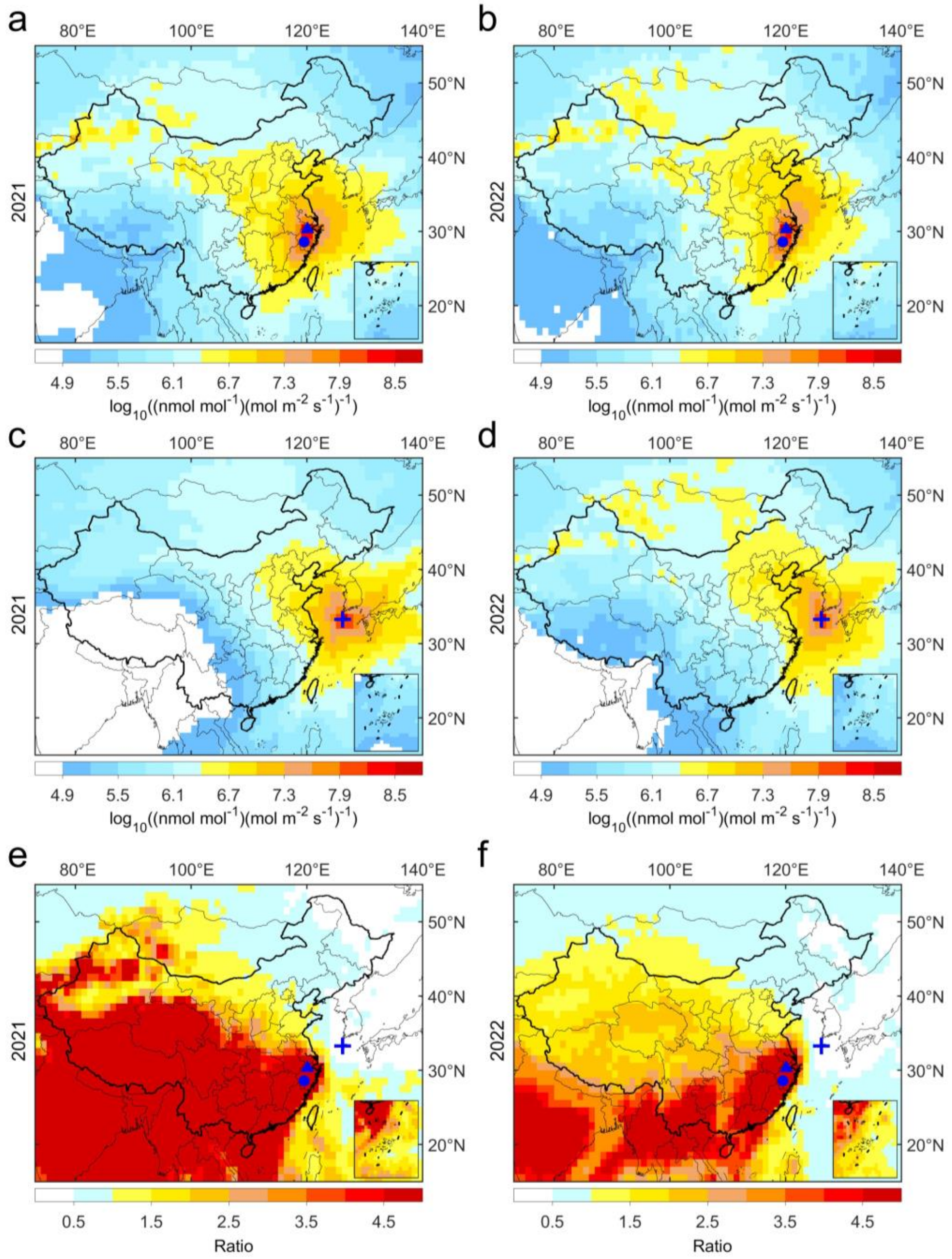

Supplementary Figure 2. Average emission sensitivity derived from FLEXPART simulations and ratios between different sites in 2021 and 2022. a, b Average emission sensitivities for ZJU (blue triangle) and SHH (blue dot) sites. **c, d** for GSN (blue cross) site. **e, f** Ratio of the average emission sensitivity of ZJU and SHH stations to that of the GSN site. The area framed by the bold black line is the target area (provincial names shown in Supplementary Fig. 1) for the inversion. ZJU, Zhejiang University; SHH, Shanghuang; GSN, Gosan.

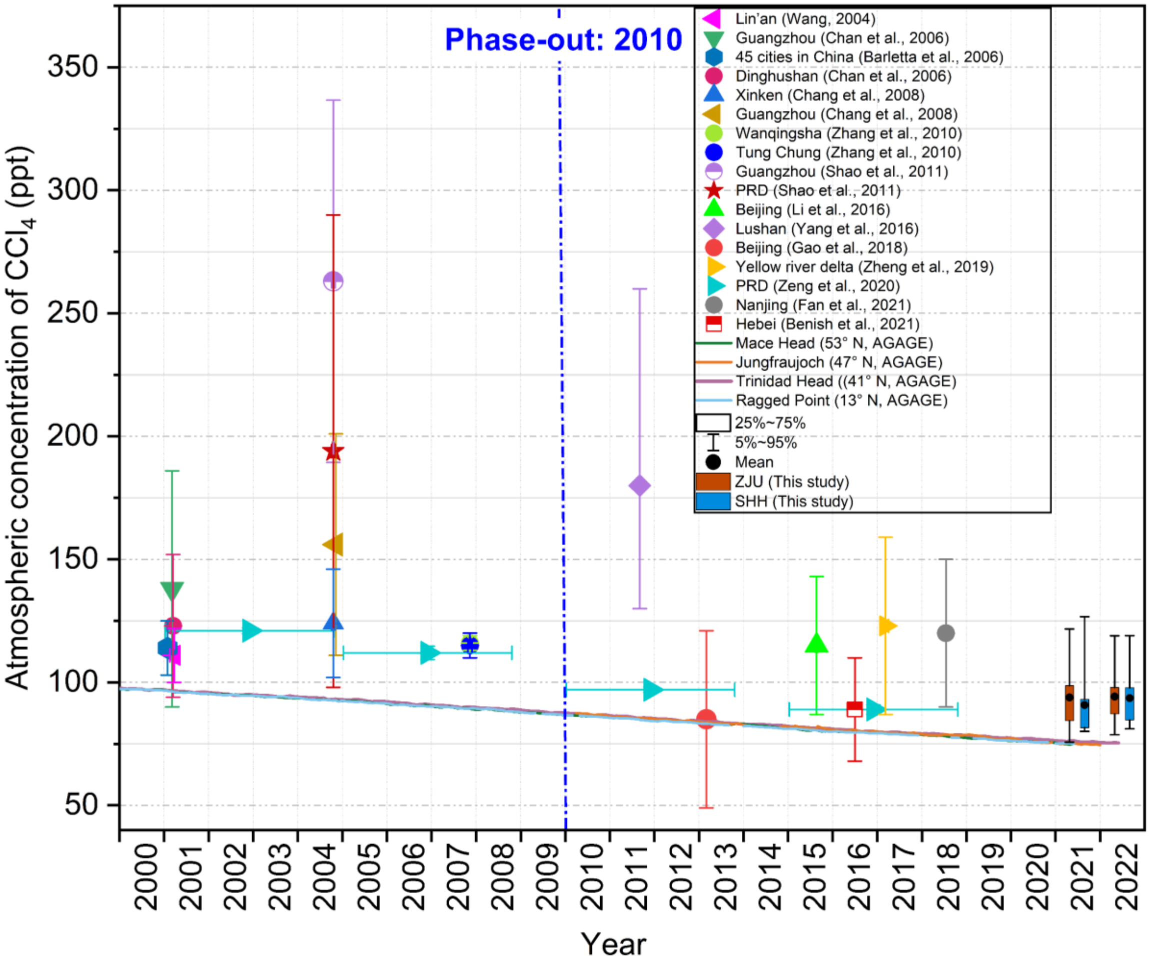

Supplementary Figure 3. Concentration levels of CCl_4 in different studies.
Uncertainties (±1 standard deviation) are represented with error bars. The concentrations of CCl_4 at ZJU and SHH are shown with box and whisker plots, the 5th, 25th, 75th, 95th percentiles and mean levels are shown. ZJU, Zhejiang University; SHH, Shanghuang.

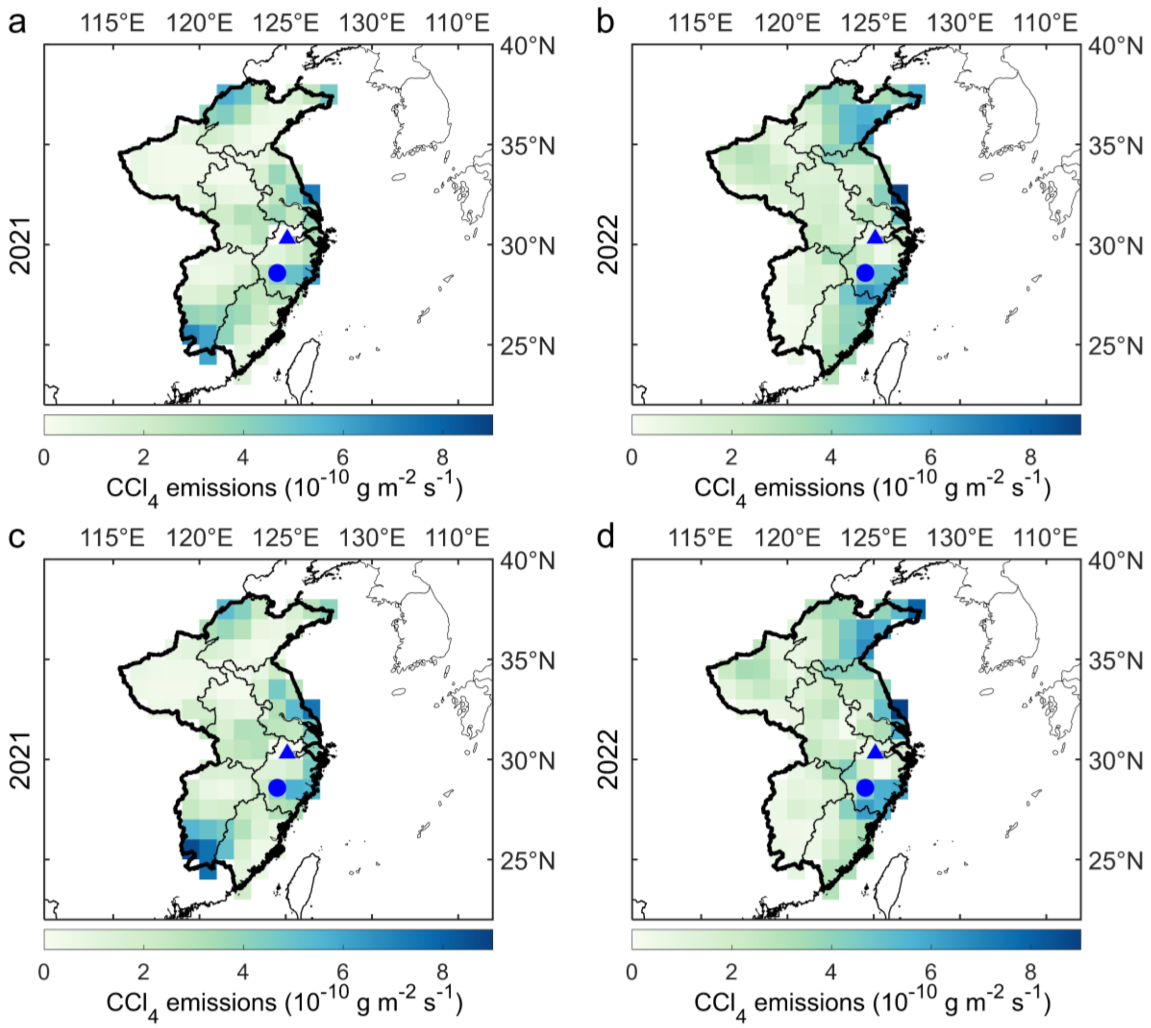

Supplementary Figure 4. Spatial distribution of CCl_4 emissions in eastern China derived from uniformly distributed prior emissions. a, b Based on baseline extracted with REBS method. **c, d** same as **a, b,** but the baseline is extracted with the method used by AGAGE. The blue triangle and dot represent the ZJU and SHH sites, respectively. REBS, robust extraction of baseline signal method; AGAGE, Advanced Global Atmospheric Gases Experiment; ZJU, Zhejiang University; SHH, Shanghuang.

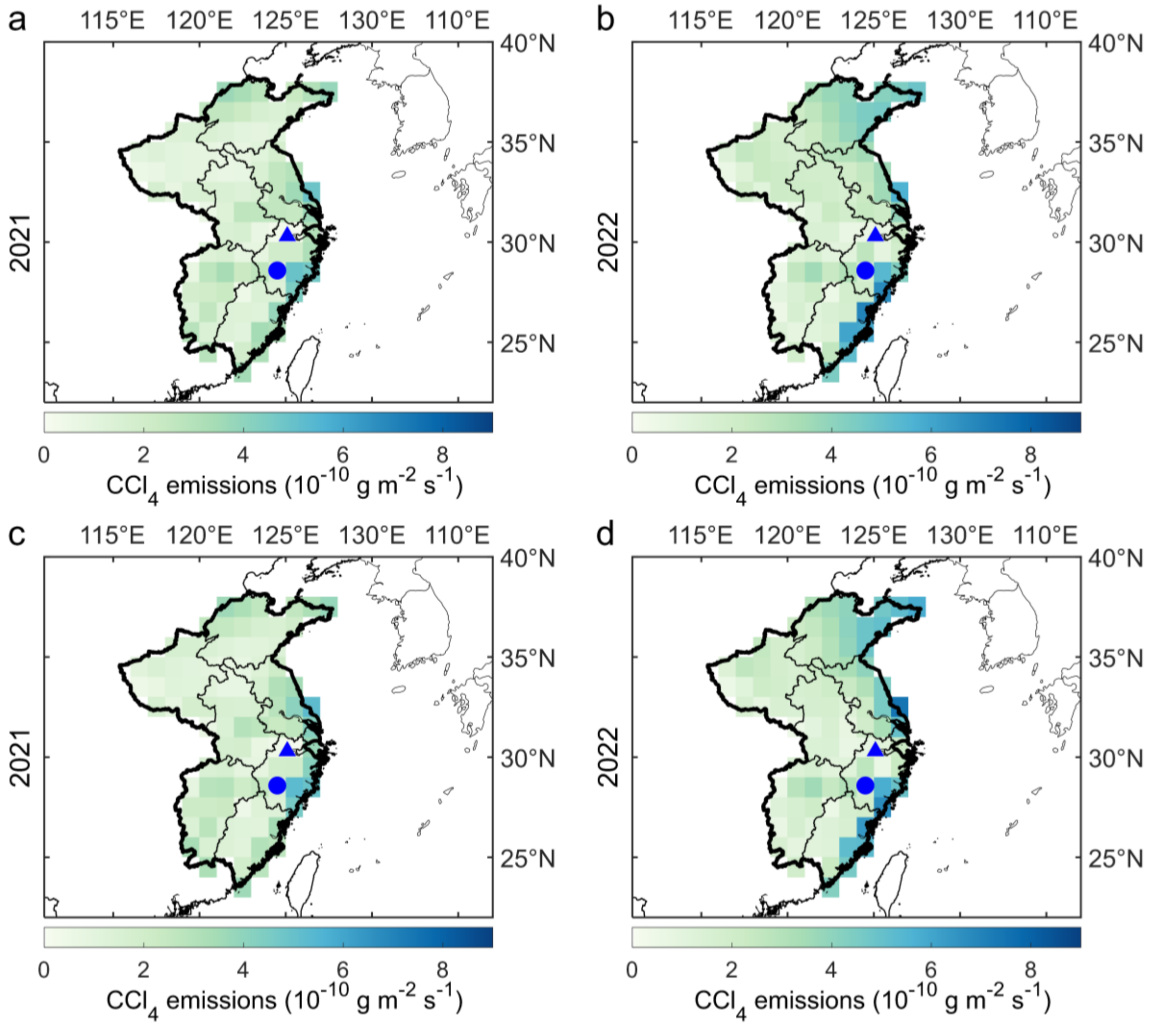

Supplementary Figure 5. Spatial distribution of CCl_4 emissions in eastern China derived from population-proxy distributed prior emissions. a, b Based on baseline extracted with REBS method. **c, d** same as **a, b,** but the baseline is extracted with the method used by AGAGE. The blue triangle and dot represent the ZJU and SHH sites, respectively. REBS, robust extraction of baseline signal method; AGAGE, Advanced Global Atmospheric Gases Experiment; ZJU, Zhejiang University; SHH, Shanghuang.

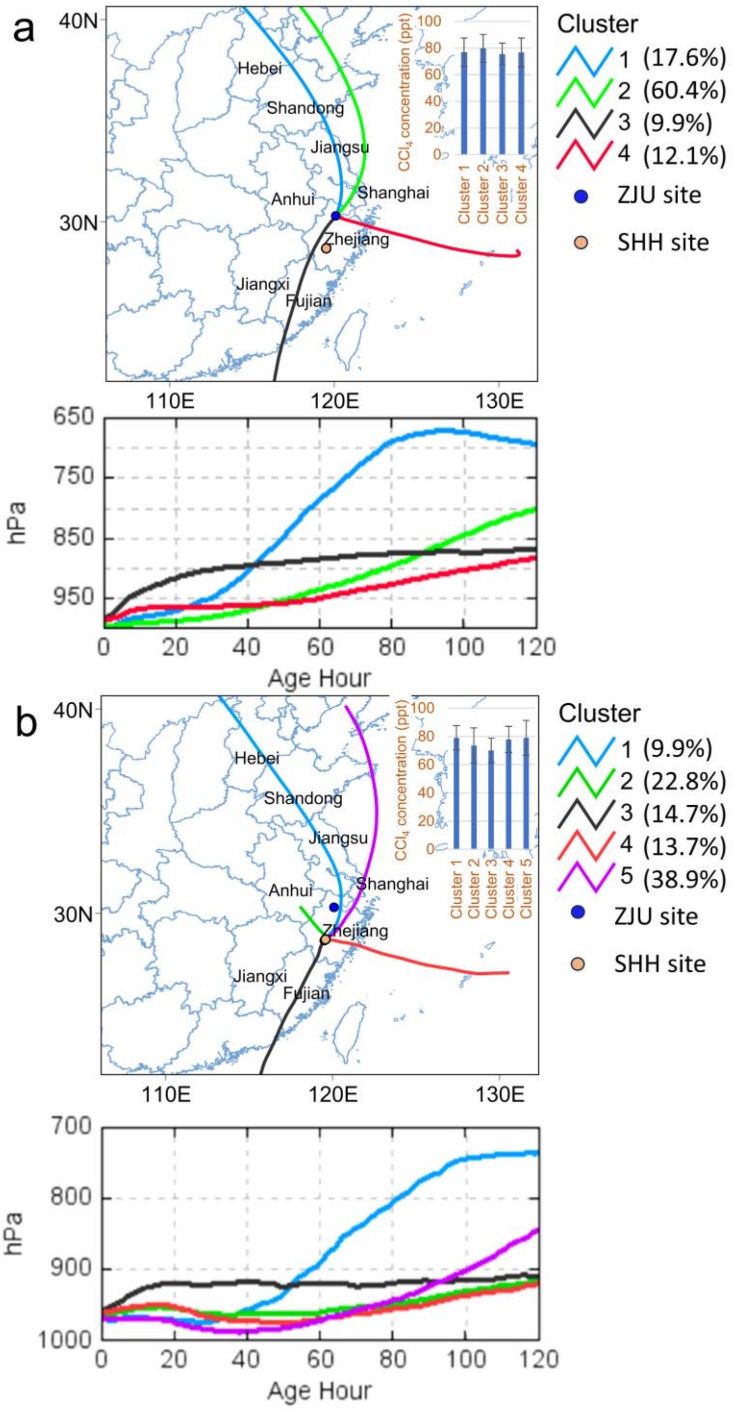

Supplementary Figure 6. Clusters of backward trajectories during the sampling period and corresponding CCl_4 concentration (error bars represent ±1 standard deviation). a Cluster analysis of 120 h backward trajectories for ZJU during the sampling period calculated using the HYSPLIT model, with the starting height at 100 m above ground level. Running intervals were set as 1 h for each day; the ratio, moving height, and average concentrations of CCl_4 of each cluster are also presented. **b** Same as **a**, excluding the SHH station. The blue and pink dots represent the ZJU and SHH sites, respectively. ZJU, Zhejiang University; SHH, Shanghuang.

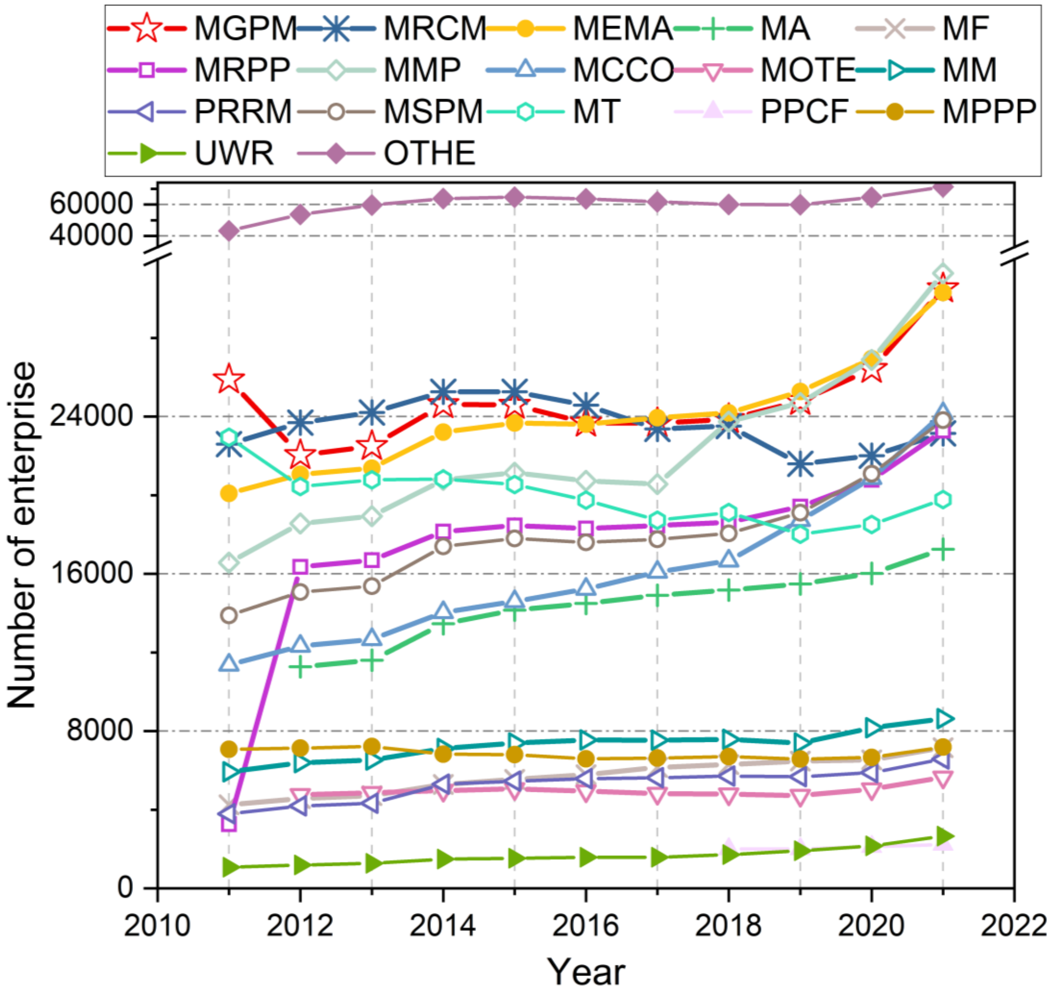

Supplementary Figure 7. Number of enterprises above designated size (annual revenue > 20 million CNY) for each industry[13]. The full name of each industrial sector is shown in Supplementary Table 2.

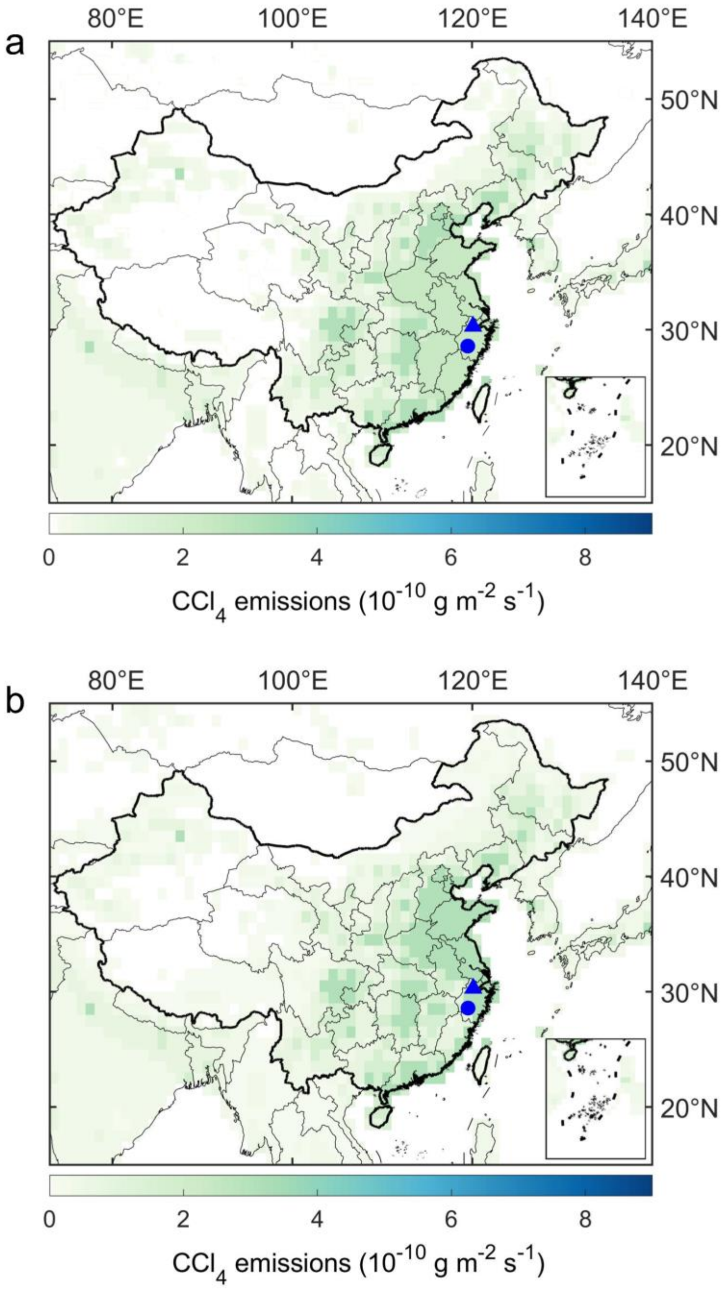

Supplementary Figure 8. CCl_4 prior emissions used in this study. a Prior emissions in eastern China set to a uniform distribution. **b** Prior emissions in eastern China distributed based on population density. The blue triangle and dot represent the ZJU and SHH sites, respectively**.** ZJU, Zhejiang University; SHH, Shanghuang.

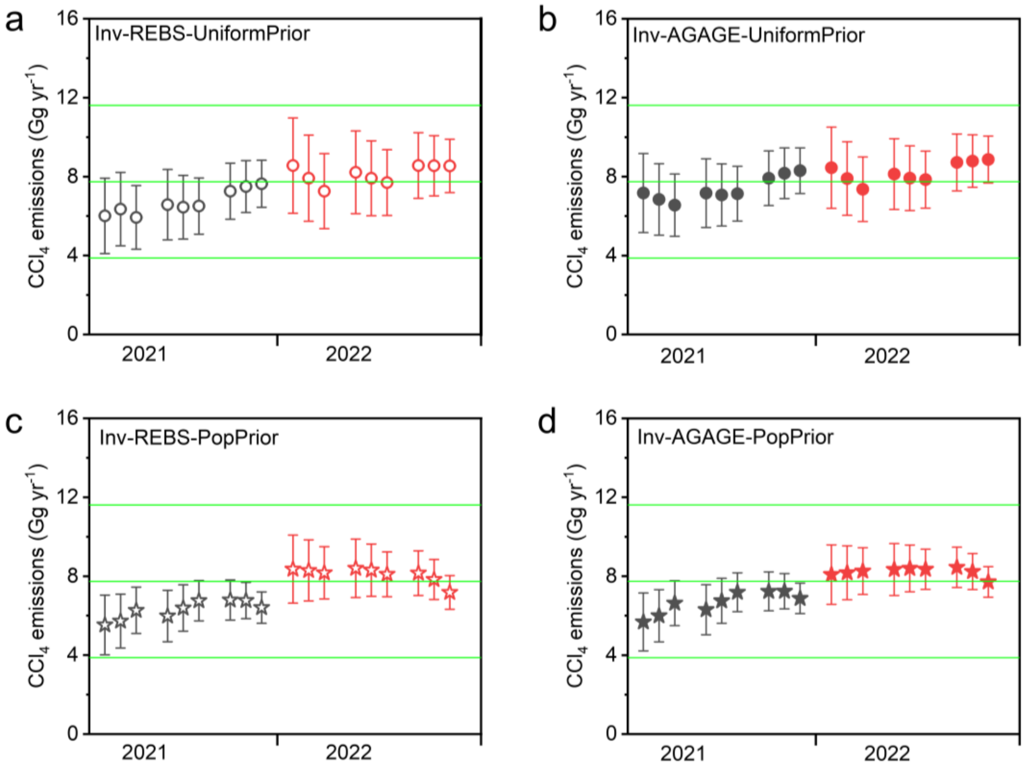

Supplementary Figure 9. Posterior CCl_4 emissions in eastern China. a Nine inversions (three magnitudes of 150%, 100%, and 50% of reference prior emission multiplied by the three magnitudes of 600%, 450%, and 300% of reference prior emission uncertainty) for CCl_4 emissions in eastern China in 2021 and 2022 based on the baseline extracted with the REBS method and uniformly distributed prior emissions (Inv-REBS-UniformPrior). **b** Same as **a** but based on the baseline extracted with the method suggested by AGAGE (Inv-AGAGE-UniformPrior). **c** Same as **a** but based on the non-uniformly distributed prior emissions (Inv-REBS-PopPrior). **d** Same as **c** but based on the baseline extracted with the method suggested by AGAGE (Inv-AGAGE-PopPrior). The three green horizontal solid lines represent three magnitudes of 150%, 100%, and 50% of reference prior emissions for eastern China. Error bars denote uncertainty range. REBS, robust extraction of baseline signal method; AGAGE, Advanced Global Atmospheric Gases Experiment.

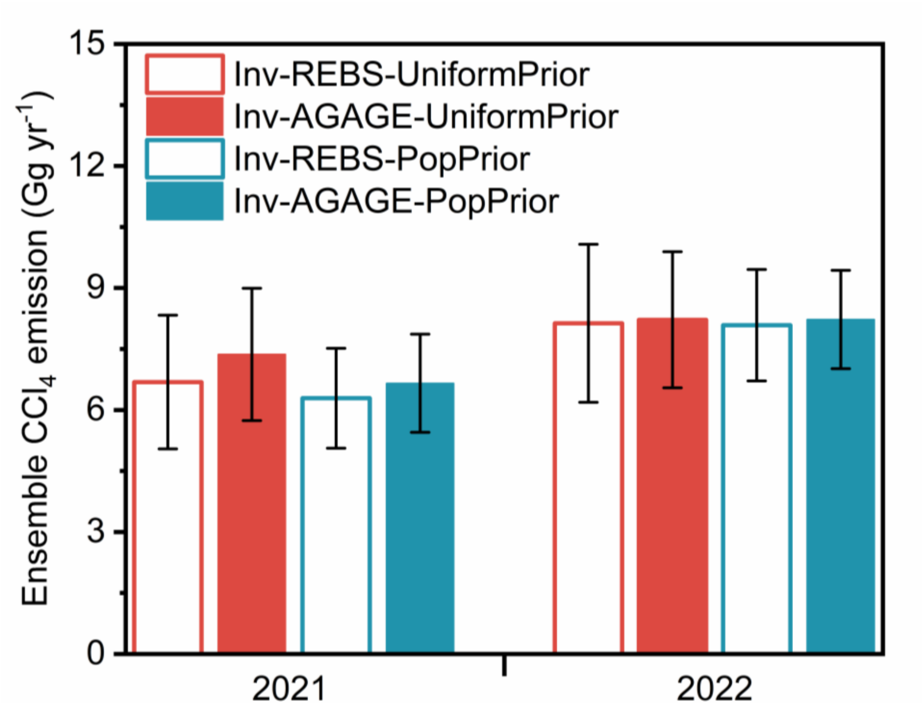

Supplementary Figure 10. CCl_4 emissions from four inversion sets for eastern China. Detailed information on four inversion sets using two baselines (AGAGE and REBS methods) and two prior emission fields (uniform and population-proxy distributions) is shown in Supplementary Table 3. Error bars denote uncertainty range. REBS, robust extraction of baseline signal method; AGAGE, Advanced Global Atmospheric Gases Experiment.

Supplementary References

1 Prinn, R. G. et al. History of chemically and radiatively important atmospheric gases from the Advanced Global Atmospheric Gases Experiment (AGAGE). *Earth Syst. Sci. Data* **10**, 985-1018 (2018).

2 Hu, L. et al. Continued emissions of carbon tetrachloride from the United States nearly two decades after its phaseout for dispersive uses. *Proc. Natl. Acad. Sci. U. S. A.* **113**, 2880-2885 (2016).

3 Fang, X. et al. Ambient mixing ratios of chlorofluorocarbons, hydrochlorofluorocarbons and hydrofluorocarbons in 46 Chinese cities. *Atmos. Environ.* **54**, 387-392 (2012).

4 Benish, S. E., Salawitch, R. J., Ren, X., He, H. & Dickerson, R. R. Airborne Observations of CFCs Over Hebei Province, China in Spring 2016. *J. Geophys. Res-Atmos.* **126** (2021).

5 Ruckstuhl, A. F. et al. Robust extraction of baseline signal of atmospheric trace species using local regression. *Atmospheric Measurement Techniques* **5**, 2613-2624 (2012).

6 Li, J. et al. Effects of rigorous emission controls on reducing ambient volatile organic compounds in Beijing, China. *Sci. Total Environ.* **557-558**, 531-541 (2016).

7 Fan, M. Y. et al. Source apportionments of atmospheric volatile organic compounds in Nanjing, China during high ozone pollution season. *Chemosphere* **263**, 128025 (2021).

8 Yang, M., Wang, Y., Chen, J., Li, H. & Li, Y. Aromatic Hydrocarbons and Halocarbons at a Mountaintop in Southern China. *Aerosol Air Qual. Res.* **16**, 478-491 (2016).

9 Zheng, P. et al. Characteristics and sources of halogenated hydrocarbons in the Yellow River Delta region, northern China. *Atmos. Res.* **225**, 70-80 (2019).

10 Stein, A. F. et al. NOAA's HYSPLIT Atmospheric Transport and Dispersion Modeling System. *Bulletin of the American Meteorological Society* **96**, 2059–2077 (2015).

11 Wang, Y. Q., Zhang, X. Y. & Draxler, R. R. TrajStat: GIS-based software that uses various trajectory statistical analysis methods to identify potential sources from long-term air pollution measurement data. *Environ. Model. Software* **24**, 938-939 (2009).

12 Sirois, A. & Bottenheim, J. W. Use of backward trajectories to interpret the 5-year record of PAN and O3 ambient air concentrations at Kejimkujik National Park, Nova Scotia. *J. Geophys. Res-Atmos.* **100**, 2867-2881 (1995).

13 National Bureau of Statistics (NBS). *China Statistical Yearbook*. (China Statistics Press, 2011-2021).
